# Supplementary material for: Genetics of Germination and Seedling Traits under Drought Stress in a MAGIC Population of Maize
Source: Plants (Basel). 2021 Aug 27;10(9):1786. doi: 10.3390/plants10091786 (PMC8468063; doi:10.3390/plants10091786)
Supplement: Supplementary file 1 [file plants-10-01786-s001.zip › plants-1340001-supplementary.pdf]

Table S1. Candidate genes detected under water stress conditions with their implications in previous studies					
Trait	Gene	Gene function	Pathway	Reference	Implication
CDW	Zm00001d035041	Phospholipase SGR2		(Fox et al. 2020)	Zm00001d035041 is a Potential ZmPIP2;5 interacting protein involved in trafficking.
CDW	Zm00001d035053	mads45 - MADS-transcription factor 45		(Vendramin et al. 2020)	Transcription factor (TF) upregulated in maize in response to ABA (abscisic acid).
CL/RL	Zm00001d037073	hct3 hydroxycinnamoyltransferase3	phenylpropanoid biosynthesis	(Wang et al. 2019)	A gene implicated in the phenylpropene (K00940) and flavonoid (K00941) metabolic pathways, encoding the down-stream enzymes of phenylalanine metabolism it was down-regulated, thereby inhibiting the degradation of phenylalanine.
CL/RL	Zm00001d037240	dhar4 - glutathione dehydroascorbate reductase4		López-Malvar et al. 2020)	This gene is suggested as a probable candidate for the QTL detected for ADF (acid detergent fiber) a forage digestibility trait.
CL/RL	Zm00001d037278	diphosphate:fructose-6-phosphate 1-phosphotransferase	glycolysis IV (plant cytosol)	(Wang et al.2019)	A gene involved in in carbohydrate metabolism, it was up-regulated.
CDW	Zm00001d003749	Aluminum-activated malate transporter 8		Gonçalves et al.2020)	Orthologue of AT1G18420 in Arabidopsis thaliana, involved in local and systemic

					inorganic phosphate sensing mechanisms in roots.
CDW	Zm00001d013415	Receptor-like kinase TMK2		Zhang et al.2020a)	Candidate tolerance gene to low-temperature during maize germination.<br>Associated with RGL: Relative germination length and RSVI: Relative simple vitality index.
CDW	Zm00001d013420	P-loop containing nucleoside triphosphate hydrolase superfamily protein		(Liu et al. 2019)	Identified as candidate nicosulfuron sensitivity gene (Nss) in maize.
CDW	Zm00001d018366	emb17 - embryo defective17		(Watkins et al. 2020)	Orthologue of AT2G24060 in *Arabidopsis thaliana*, protein associated with the mRNA encoding the D1 reaction center protein of Photosystem II in plant chloroplasts, and an essential gene for maize kernel development.
CDW	Zm00001d018401	ptac17 - plastid transcriptionally active17		(Watkins et al. 2020)	Orthologue of AT1G80480 in Arabidopsis thaliana, protein associated with the mRNA encoding the D1 reaction center protein of Photosystem II in plant chloroplasts.

CDW	Zm00001d038608	gras62 - GRAS-transcription factor 62		(Su et al. 2014)	A gene associated with (average cortical cell size in the whole root cortex) CCS under well-watered conditions was found to be upregulated in maize grown with low Phosphorus (P)..
CDW	Zm00001d012097	Pentatricopeptide repeat-containing protein		(Liang et al. 2019)	It' s a PPR gene (pentatricopeptide repeat gene) with biological functions highly related to post-transcriptional regulation in mitochondria and chloroplasts (Barkan and small, 2014)
CDW	Zm00001d012159	glu4 - beta-glucosidase4		(Sekhon et al. 2019)	Orthologous to Arabidopsis BGLU42 and rice Os1bglu4, The b-glucosidaseactivity increases during Arabidopsis leaf senescence it may have a role in sugar signaling.
CDW	Zm00001d012170	Calreticulin-3		(Hu et al. 2020)	A gene involved in proteins metabolism
RDW	Zm00001d039635	bzr6 - BZR-transcription factor 6		(Liu et al. 2018)	Belongs to BES1 gene family, BES1 transcription factors are critical for BR (Brassinosteroids (BRs), signal transduction. Brassinosteroids (BRs), which are essential

					phytohormones for plant growth and development.
RDW	Zm00001d025764	Endoglucanase 7		(Yao et al. 2020)	Candidate genes for resistance to Fusarium ear rot in maize.
CDW/RDW	Zm00001d029702	Glutathione S-transferase U16		(Pinto et al. 2019)	A GST gene down regulated after aluminum stress, GST might play a role in maize roots alleviation against Aluminum toxicity.
CDW/RDW	Zm00001d029715	alpha/beta-Hydrolases superfamily protein		(Griffiths et al. 2020)	A gene involved in the response to abiotic stimulus.
CDW/RDW	Zm00001d007188	ETHYLENE INSENSITIVE 3-like 2 protein		(Vendramin et al. 2020)	Transcription factor (TF) downregulated after ABA induction.
CDW/RDW	Zm00001d007190	Zinc finger (C3HC4-type RING finger) family protein		Li et al. 2020a)	This gene showed low expression levels at each stage of seed formation in maize.
CDW/RDW	Zm00001d007191	myb110 - MYB-transcription factor 110		(Li et al. 2020a)	This gene showed low expression levels at each stage of seed formation in maize.
CDW/RDW	Zm00001d007194	Calmodulin-7		(Li et al. 2020a)	This gene was highly expressed during the whole period of embryo and endosperm development in maize.

CDW/RDW	Zm00001d007196	dihydrofolate reductase	folate transformations II	(Li et al. 2020a)	Potential functional gene significantly associated with ETB (ear tip-barrenness in maize).
CDW/RDW	Zm00001d007207	putative protein phosphatase 2C 5		(Li et al. 2020a)	This gene showed low expression levels at each stage of seed formation in maize.
CDW/RDW	Zm00001d007242	Thioredoxin superfamily protein		(Sandhu et al. 2020)	A candidate gene associated with salt tolerance in maize. Significantly associated with RW/SW root weight/shoot weight ratio.
CDW/RDW	Zm00001d007267	lhcb5 - light harvesting chlorophyll a/b binding protein5		(Li et al. 2020b)	Candidate gene identified as possible hub gene involved in temperature stresses and was mainly involved in photosynthesis.

Table S2. Candidate genes detected under well-watered conditions with their implications in previous studies					
Trait	Gene	Gene function	Pathway	Previous studies	Implication
G%	Zm00001d049187	6-phosphogluconate dehydrogenase, decarboxylating	pentose phosphate pathway (oxidative branch) I	(Peng et al.2020)	Candidate gene for CRN (Crown root number), in a limited nitrogen availability.
G%	Zm00001d049191	2,3-diketo-5-methylthio-1-phosphopentane enolase	S-methyl-5-thio-α-D-ribose 1-phosphate degradation	(Sekhon et al. 2019)	Candidate gene associated with leaf senescence in maize.
CL	Zm00001d038717	lbd33 - LBD-transcription factor 33		(Frey et al.2020)	Gene expressed under cold and control conditions in maize, and up-regulated at control.
CL/RL	Zm00001d002723	mlkg1 - Maize LINC KASH Grass-specific1		(Gumber et al. 2019)	Is a maize gene encoding (LINC) The linker of nucleoskeleton to cytoskeleton (LINC) complex. It connects the cytoplasm to the nucleoplasm, functions to maintain nuclear

					shape and architecture and regulates chromosome dynamics during cell division.
CL/RL	Zm00001d042471	General transcription factor 2-related zinc finger protein		(Wang et al. 2019)	Gene Involved in Lysine Metabolism which was upregulated after pollination.
CDW	Zm00001d021177	hypothetical protein		(Watkins et al.2020)	Orthologue of AT1G21500 in Arabidopsis thaliana.
CL/RL	Zm00001d021952	Calcium-dependent lipid-binding (CaLB domain) family protein		(Hu et al. 2020)	Involved in protein metabolism
CL/RL	Zm00001d011953	arftf25 - ARF-transcription factor 25		Chen et al. 2017)	Candidate gene for the major QTL conferring resistance to GSR (Gibberella stalk rot (GSR), it encodes an auxin response factor.
CL/RL	Zm00001d012535	Putative bifunctional inhibitor/LTP/seed storage protein family		(Castorina et al.2020)	This gene has a role in binding and transport of the fatty acid, cutin and wax monomers and candidate gene involved in lipid metabolism for cuticle biosynthesis in maize.

CL/RL	Zm00001d012544	myb74 - MYB-transcription factor 74		(Li et al. 2019)	Belongs to the TCP family. Zm00001d012544, maize orthologue of OsGAMYB in rice which has essential functions in floral organ and pollen development in rice. Additionally, Zm00001d012544 is highly expressed in the maize anther suggesting that may be an important regulator of maize anther development.
CL/RL	Zm00001d012640	Jacalin-related lectin 3		(Liu et al. 2019)	Potential candidate gene involved in maize kernel size traits.
